# Infarct in new territory after endovascular stroke treatment: A diffusion-weighted imaging study

**DOI:** 10.1038/s41598-020-64495-2

**Published:** 2020-05-20

**Authors:** Johannes Kaesmacher, Christoph Kurmann, Noel Jungi, Philipe Breiding, Matthias F. Lang, Raphael Meier, Tomas Dobrocky, Eike Piechowiak, Felix Zibold, Sebastian Bellwald, Thomas R. Meinel, Mirjam R. Heldner, Pasquale Mordasini, Marcel Arnold, Pascal J. Mosimann, Mayank Goyal, Jan Gralla, Urs Fischer

**Affiliations:** 1 University Institute of Diagnostic and Interventional Neuroradiology, University Hospital Bern, Inselspital, University of Bern, Bern, Switzerland; 2 Department of Neurology, University Hospital Bern, Inselspital, University of Bern, Bern, Switzerland; 3 Department of Diagnostic, Interventional and Pediatric Radiology, University Hospital Bern, Inselspital, University of Bern, Bern, Switzerland; 4 Support Center for Advanced Neuroimaging - Institute for Diagnostic and Inter-ventional Neuroradiology, University Hospital Inselspital and University of Bern, Bern, Switzerland; 5 Department of Diagnostic Imaging, University of Calgary, Calgary, Alberta Canada

**Keywords:** Pathology, Outcomes research, Stroke, Risk factors

## Abstract

Data on infarcts in new territory (INT) in patients undergoing endovascular stroke treatment for acute large-vessel occlusions are sparse. Aim of this study was to assess the prevalence, risk factors, and clinical relevance of INT. For this purpose, all patients in a single-center prospective registry who underwent endovascular stroke treatment and received pre- and post-interventional diffusion-weighted imaging were included (N = 259). Using an established scoring system, INT were classified according to size (I-III, ≤2 mm, >2 mm ≤20 mm, >20 mm) and likelihood of being related to the intervention (A, high likelihood; B, low likelihood). Additionally, a new type of infarct, that occurred in a territory distal to the occlusion, but was initially not hypoperfused, was defined as an infarct in initially not hypoperfused territory (IINHT). A total of 180 INT and 38 IINHT were observed in 32.8% (N = 85/259) of patients. In most patients, INT were angiographically occult (90.2%), and 13 patients had INT/IINHT larger than 2 cm (type III). Absence of protection during stent-retrieval and a cardio-embolic stroke origin were associated with higher incidence of INT/IINHT, whereas pretreatment with IV tPA showed no association, even when different bolus timing was considered. INT/IINHT were associated with lower rates of functional independence with increasing size type after adjusting for confounders **(**adjusted Odds Ratio per size group increase 0.63, 95% confidence interval 0.46–0.86). In conclusion, INT and IINHT are not rare, are associated with poor outcome with increasing size, and they may serve as a surrogate endpoint for safety evaluation of new devices and endovascular techniques. Further research on associated factors is warranted.

## Introduction

Infarct in a new territory (INT), which was initially not affected, is a potentially severe complication of endovascular stroke treatment (EVT), and is associated with poor outcome^1–5^. Embolic complications can be detected on procedural or post-procedural angiography images, and are referred to as emboli in a new territory (ENT)^4,5^. Alternatively, they are found by evaluating new ischemic lesions on follow-up imaging, and are referred to as INT^2,3,5^. The latter approach is more sensitive for detecting non-apparent emboli, since it is able to detect very small infarcts and infarcts occurring in territories that are not made visible by procedural angiography (e.g. contralateral hemisphere emboli originating from the aortic arch).

In the ESCAPE trial, around 10% of patients were found to have INT, based on post-procedural MRI including diffusion-weighted imaging (DWI). The majority of these INT were in a pattern incompatible with a guide catheter origin^3^. Preliminary evidence from this and one other study suggested that pretreatment with intravenous thrombolysis (IVT) may offer protection from INT^6^. Other studies, however, failed to reproduce this association when considering ENT^4,5^. A common challenge in defining INT is that multiple occlusions or small infarcts unrelated to the initially occluded territory may be present even before the intervention starts, potentially accompanying the pathology of the index stroke (e.g. disintegration of an unstable large artery or cardiogenic clot)^3,7^. In order to define new infarcts that are likely associated with the procedure an effective detection of infarcts before and after the procedure is therefore essential.

The aim of this study was to describe the frequency, types and severity of INT according to recently proposed criteria^2^. These criteria were applied in a cohort of patients with acute ischemic stroke who underwent endovascular treatment together with pre- and early post-interventional MRI including DWI. Secondary objectives included the evaluation of associated factors and the clinical relevance of INT and their subtypes.

## Methods

### Study cohort and follow-up

All patients with intended endovascular treatment between January 2012 and August 2017 were screened for this study. Patients were included if their pre-interventional diagnostic work-up consisted of an MRI with DWI sequences and if they had undergone a follow-up MRI within 80 h after the intervention (median delay after the intervention: 24 h, interquartile range 20–27 h, 55.2% ≤ 24 h, 92.7% ≤ 48 h, 98.5% ≤ 72 h). Clinical and baseline characteristics of patients were extracted from the prospective Bernese Stroke Registry, as described previously^7^. Ethical approval for this retrospective analysis was obtained from the local ethics committee (Kantonale Ethikkommission für die Forschung Bern, Bern, Switzerland, amendment access number: 231/2014) and data collection was performed in accordance with relevant guidelines and regulations. All patients gave their informed consent or consent was waived as per institutional and ethics committee regulations, depending on the time of inclusion in the registry. For the prospective Bernese Stroke Registry, clinical functional outcome (modified Rankin Scale, mRS) is assessed at day 90 during a routinely scheduled clinical follow-up or – if the patient is unable to attend – by a trained and mRS-certified study nurse applying a standardized telephone interview. Favorable outcome was defined as modified Rankin Scale (mRS) ≤2. Neurological worsening was defined as an increase in the NIHSS score of ≥4 points.

### Classification of INT and image analysis

**Figure 1:**
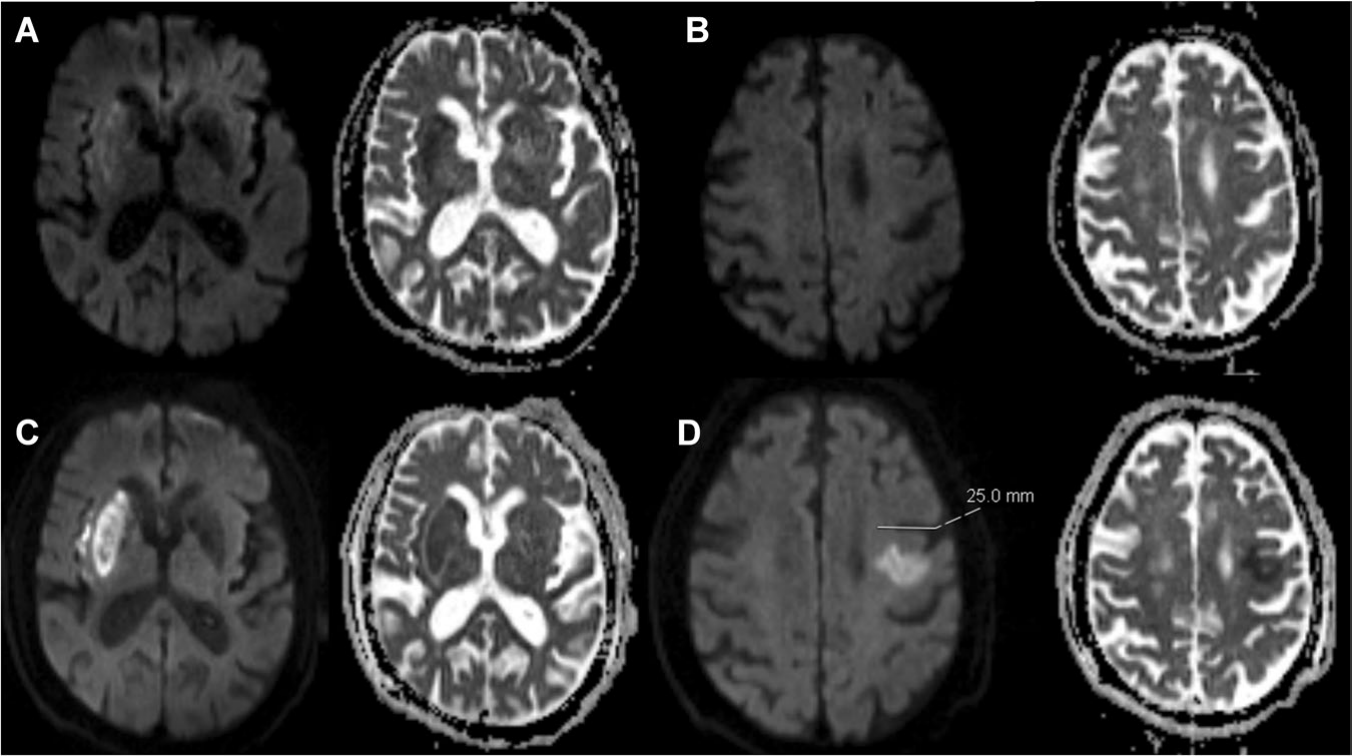
Large infarct in new territory without catheter manipulation across the ostium (type IIIB). (**A**,**B)** preinterventional diffusion-weighted imaging/apparent diffusion coefficient revealed mild diffusion restriction within the striatocapsular region and insular cortex on the right-hand side. No diffusion restriction in the contralateral hemisphere is delineable. (**C**,**D)** postinterventional DWI/ADC revealed marked diffusion restriction in the right middle cerebral artery territory without substantial infarct growth; however, a new DWI/ADC lesion is seen in the contralateral hemisphere in close proximity to the precentral sulcus, corresponding to a size type III (>20 mm), manipulation type B (the catheter was not manipulated past the ostium of the new territory) infarct in new territory.

**Figure 2:**
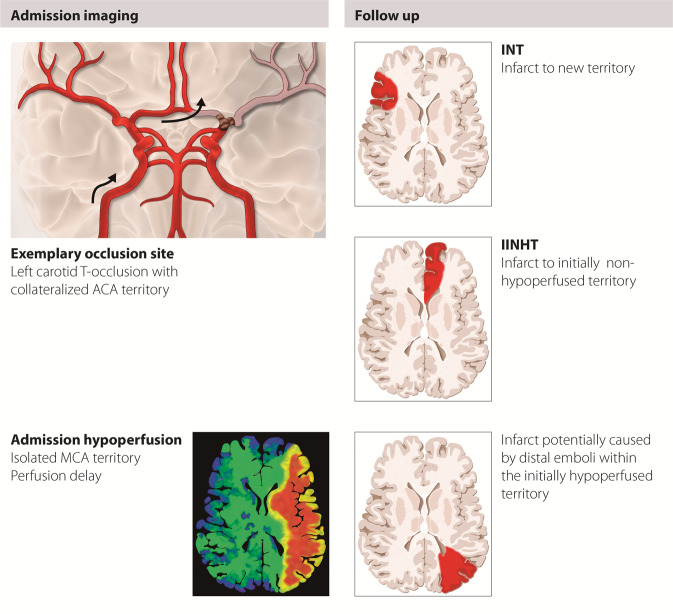
Schematic representation of infarcts in new territory (INT) and infarcts in initially not hypoperfused territory (IINHT). Illustration by Anja Giger (Inselspital Bern, Bern, Switzerland).

Classification of INT was performed according to the methodology proposed by Goyal *et al*.^2^. For this purpose, pre-procedural non-invasive imaging, post-procedural angiography images, and follow-up scans were reviewed using standardized data presentation and worksheets (Supplementary Figure e1 and Supplementary Table e1). INT was defined as a ‘new’ DWI lesion (i.e. not delineated on pre-procedural non-invasive imaging) outside the affected territory (every territory that is not located distal to the clot). Each INT was then classified according to its size (types I-III, ≤2 mm, >2 mm - ≤20 mm, >20 mm, respectively) and likelihood of being related to the procedure: A - catheter was manipulated past the ostium of the new territory or B - catheter was not manipulated past the ostium of the new territory, see Supplementary Table e2 for further details)^2^. Classification according to catheter manipulation depended on the placement of the guiding/balloon guide catheter and/or distal aspiration catheter. In the present study, size classification was based on axial B1000 images using the largest diameter of the lesion. An example of a size type III manipulation type B INT is shown in Fig. 1. The following amendments to the classification proposed by Goyal *et al*. were made: (1) Number of INT was recorded, and, for each INT, size and type was determined; (2) If DWI showed a new lesion within the affected territory, but outside the initially hypoperfused area, as determined from pre-procedural MR perfusion imaging, this was considered as an infarct in initially not hypoperfused territory (IINHT, see schematic overview in Fig. 2 and example case in Supplementary Figure e2). Additionally, the occurrence of DWI lesions outside the occluded territory on admission imaging and the presence of multiple occlusions and the respective distinct hypoperfused areas were recorded (Supplementary Figure e3)^7^. Pre- and post-interventional MRIs were acquired using a 1.5/3T scanner (Magnetom Avanto or Magnetom Verio, Siemens, Erlangen, Germany). The institutional MRI protocol consisted of fluid-attenuated inversion recovery, DWI, susceptibility-weighted imaging, contrast-enhanced cervical angiography, intracranial time-of-flight angiography and a gradient-echo dynamic susceptibility contrast perfusion sequence. Initial stroke lesion size before treatment was assessed with the DWI based Alberta Stroke Program Early CT Score (DWI-ASPECTS)^8^.

All pre-interventional contrast-enhanced MR angiographs were evaluated for aortic arch elongation and branching patterns as described previously^9–11^. For this purpose, the branching pattern was classified into 3 types according to the vertical distance from the innominate artery to the apex of the aortic arch. Classification as type I, II or III was based on this distance as follows: <1 diameter of the CCA (Type I), 1–2 diameter of the CCA (Type II) or >2 diameter of the CCA (Type III)^9,11^. In addition, the aortic arch was classified according to supraaortic branching patterns. Separate origins for the innominate, left common carotid, and left subclavian arteries was defined as a normal branching pattern^10^. A bovine arch was defined as a common origin of the innominate artery and the left common carotid artery, whereas branching of the left common carotid artery originating separately from the innominate artery was classified differentely^10^. As an indicator of aortic arch atherosclerosis, any aortic arch branch stenosis>50% was recorded as proximal supraaortic branch stenosis. We did not assess aortic arch atherosclerosis and or calcification independently owing to the often poor quality of contrast-enhanced MR angiography at the caudal end of the field of view, and the lack of precision regarding the extent of calcifications when using contrast-enhanced MRA.

### Endovascular treatment

General institutional eligibility criteria for acute ischemic stroke patients receiving endovascular treatment can be found online: http://www.neurologie.insel.ch/fileadmin/neurologie/neurologie_users/Unser_Angebot/Dokumente/Stroke_Guidelines_2018_01.pdf. Of all included patients, 220 patients had stent-retriever thrombectomy, 28 were treated with intra-arterial urokinase only, one patient with manual thrombus fragmentation was treated using a microwire and in 11 patients endovascular thrombectomy was withheld after partial or complete thrombolysis was noted on the first diagnostic angiography runs. When the institutional criteria for endovascular therapy were fulfilled, digital subtraction angiography was performed via a transfemoral approach by using a biplane, high-resolution angiography system (Axiom Artis zee; Siemens, Erlangen, Germany). Pre-interventional 3-vessel angiography was routinely performed to assess cross-flow and collaterals. According to the institution’s standard operating protocols, pre-interventional diagnostic angiography has to be performed within 3 minutes; otherwise, it was discontinued, and endovascular stroke treatment was initiated. For this purpose, a 5F JB3 cerebral catheter (Cook Medical, Bloomington, Indiana, USA) was used. In 63.5% (N = 165) of patients, diagnostic angiography was performed according to the standard operating procedure protocol, while in 20% (N = 52) only the two common carotid arteries were injected, and in 13.1% (N = 34) only the affected territory was displayed. In the remaining cases (3.5%, N = 9), one vertebral artery and one common carotid artery was injected. Stent-retriever-based thrombectomy was usually performed in conjunction with an 8F or 9F balloon-guide catheter (Merci Balloon Guide Catheter, Concentric Medical, Mountain View, California, USA; FlowGate Balloon Guide Catheter, Stryker, Kalamazoo, Michigan, USA). The catheter was placed in the cervical ICA using a long exchange wire (Supra Core Guide Wire, Abbott, Chicago, Illinois, USA) after diagnostic angiography (N = 105/220, 47.7%). If the vascular architecture of the patient was deemed unsuitable for balloon-guide catheter placement, a distal access catheter was used instead (3MAX/5MAX ACE, Penumbra, Alameda, California, USA; DAC Catheter, Stryker; AXS Catalyst, Stryker; Sofia, Microvention, Aliso Viejo, California, USA; N = 94/220, 42.7%). In a few cases, the distal access catheter was used as a second-line treatment if the initial attempt at placing a balloon-guide catheter was unsuccessful or reperfusion could not be achieved with this setup (N = 5/220, 2.3%). In the remaining patients, a balloon-guide catheter was primarily used together with a distal aspiration catheter in the same setup (N = 5/220, 2.3%). Most patients were treated using a Solitaire stent-retriever (Medtronic, Minneapolis Minnesota, USA). Intra-arterial urokinase was administered after failed intracranial access or if patients presented with distal vessel occlusions only^12^. For urokinase administration, a 6F or 8F guiding catheter was used and selective intracranial access was established using a 0.021.inch Prowler Select Plus (Codman, Boston, Massachusetts, USA) or an Excelsior SL-10 (Stryker) microcatheter. The dose of Urokinase administered varied between 250000 and 1000000 IU. Good-quality post-procedural overview runs for the evaluation of emboli in initially not hypoperfused territories were available for 253/259 (97.7%) of patients.

### Statistical analysis

Continuous and discrete numerical variables are shown as median and interquartile range. Comparisons between frequency counts were made using chi-square or Fisher´s exact test (2 × 2 contingency tables). Binary logistic regressions with dichotomized clinical outcomes as dependent variable were adjusted for the prespecified confounders age, sex, admission NIHSS, IV tPA, reperfusion success, DWI-ASPECTS and site of occlusion. For sensitivity purposes, patients were additionally divided into 3 groups: (1) patients who did not receive IV tPA, (2) patients in whom the interval between administration of the IV tPA bolus and the intervention was ≥70 minutes (=i.e. presumed low IV tPA concentration/no therapeutic concentrations) and (3) patients in whom the interval between IV tPA bolus administration and the intervention was <70 minutes (=i.e. presumed therapeutic tPA concentration). A potential effect of the time interval between intervention and post-interventional MRI on the occurrence of INT/IINHT was evaluated by comparing these metrics across INT/IINHT-positive and negative patients. Statistical analyses were performed using SPSS statistics.

## Results

### Study cohort

Two hundred fifty-nine patients met the inclusion criteria (median age on admission: 71 years, IQR 57–79 years, 49.0% female). Patients underwent endovascular treatment for severe strokes (median NIHSS 11, IQR 6–16) and mostly for anterior circulation occlusions (84.9%, N = 220). In total, 45.2% (N = 117) of patients were pretreated with IV tPA, and 69 patients (59.0.%) received an IV tPA bolus ≤70 minutes before the intervention. Successful reperfusion, defined as modified Thrombolysis in Cerebral Infarction (TICI) 2b/3, was achieved in 208 patients (80.3%). The median delay from start of intervention to follow-up MRI was 23 h (IQR 20–27 h).

### Frequency and types of INT and IINHT

**Figure 3:**
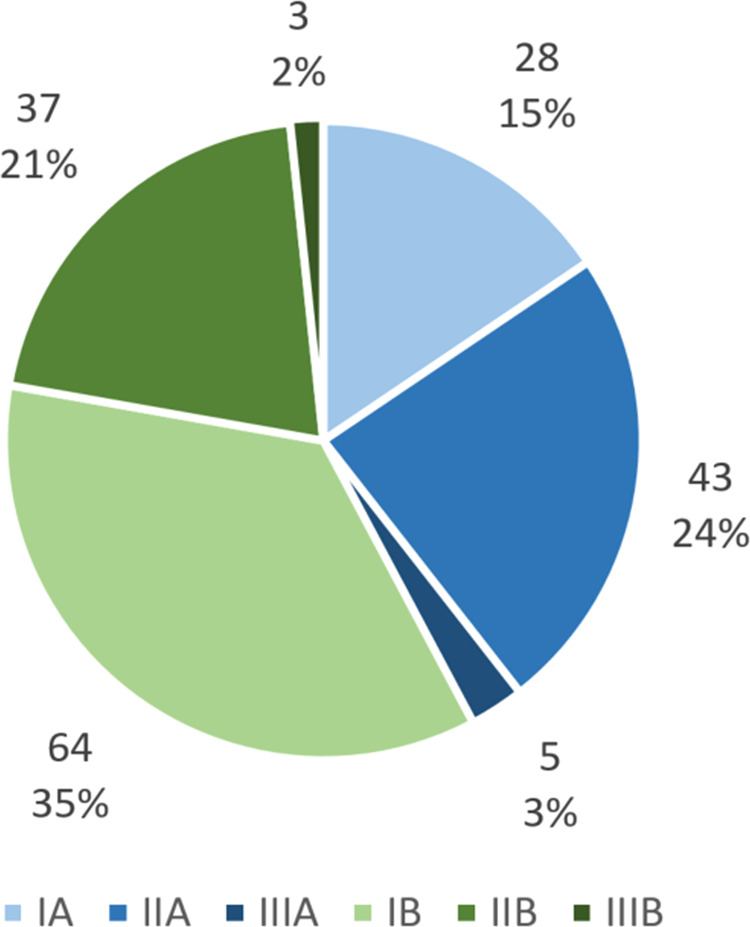
Distribution of infarcts in new territory (INT). Most INT occurred when there was no catheter manipulation across the infarcts territory ostium (manipulation type B), and were small (size type I). The most common type of INT was IB (35%). Large INT were rare (5%).

One-hundred-and-eighty INT were recorded in 76/259 (29.2%) patients. Of the 180 INT, 76 (42.2%) were categorized as type A and 104 (57.8%) as type B (Fig. 3). Relative distribution of INT according to size class was 51.1% (N = 92) type I, 44.4% (N = 80) type II and 4.4% (N = 8) type III (Fig. 3). At least one size type III INT was seen in 3.1% of patients (8/259), while 13.5% of patients (35/259) had at least one type A INT.

**Figure 4:**
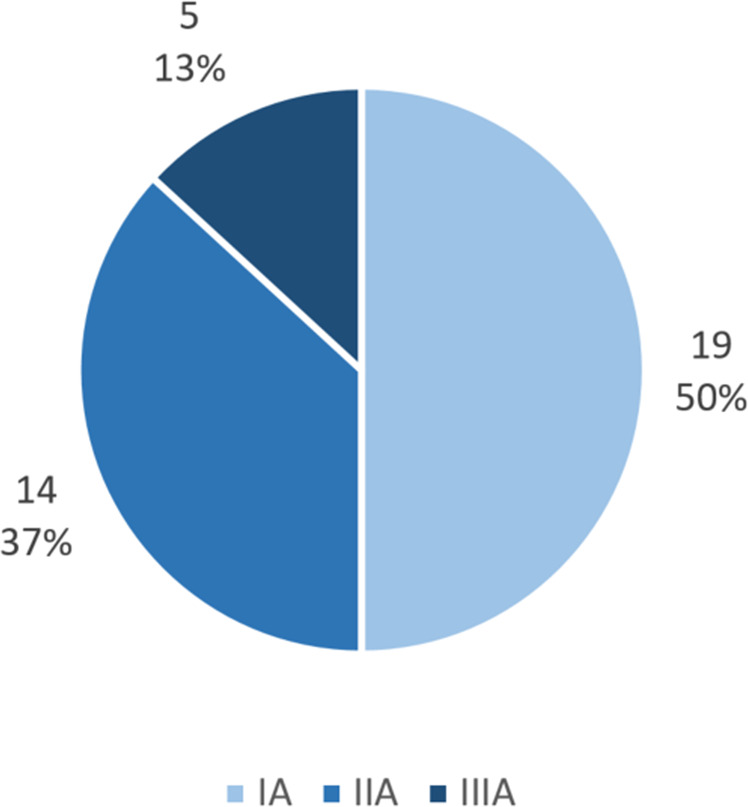
Distribution of IINHTs. Relative frequency of infarcts in initially not hypoperfused territory (IINHT) occurring in 15 patients (6 with concomitant INT). Around 1 in 10 IINHT resulted in a large infarct ≥2 cm (N = 5/38, 13%).

IINHT occurred in 15/259 (5.8%) patients (total number of IINHTs: 38). By definition, all IINHT were categorized as type A. Relative frequencies of size type I, II and III were 50.0% (N = 19), 36.8% (N = 14) and 13.2% (N = 5), respectively (Fig. 4 and Supplementary Figure e4). At least one INT or IINHT was observed in 85/259 patients (32.8%). Taking INT and IINHT together, the frequency of patients with at least one size type III, at least one size type II (but not III), and at least one size type I (but not II or III) was 4.6% (N = 12/259), 17.8% (N = 46/259), and 10.4% (N = 27/259), respectively. More than two-thirds of patients with angiographically visible emboli developed an INT or IINHT (72.7%, 8/11), while only a minority of all INT/IINHTs was angiographically detectable (9.8%, 8/82; see Supplementary Table e3 A). Only 2/12 size type III infarcts were detectable on post- or peri-procedural angiography runs (Supplementary Table e3 B). The interval between procedure initiation and follow-up imaging did not differ significantly between INT/IINHT-positive and negative patients (median 23.4 h vs 23.6 h, p = 0.998).

### Factors associated with INT/IINHT

The presence of multiple occlusions or multiple embolic infarcts on admission did not differ between patients with and without INT/IINHT (4.6% vs 5.9%, p = 0.763 and 12.6% vs 14.1%, p = 0.845, respectively, Table 1). Rates of IV tPA pretreatment did not differ between the groups (43.7% vs 48.2%, p = 0.509) and there was a tendency toward more cardioembolic strokes in patients experiencing INT/IINHTs, which reached significance in dichotomization only (p = 0.045). This association was more pronounced when evaluating INT, IINHT and those without INT/IINHT separately, showing higher rates of cardioembolic strokes in patients with INT (53.9% in INT, vs 36.2% in controls without INT or IINHTs, vs 11.1% in IINHTs, P = 0.005, Supplementary Table e4). There was a slight trend towards more INT/IINHT if only a cervical non-balloon-guide catheter was used instead of a balloon-guide catheter and/or a distal access catheter. However, other aspects of the intervention, including the location and mode of pre-interventional diagnostic angiography, did not differ between the groups. Aortic arch elongation and branching anatomy patterns did not differ between patients who developed INT/IINHT and those who did not.

**Table 1:** Comparison of patients with and without INT/IINHT.

	All (N = 259)	No INT/IINHT (N = 174)	INT/IINHT (N = 85)	P
Age (years)	71.0 (58.0–79.1)	69.3 (57.5–78.9)	72.0 (60.3–80.0)	0.200
Sex, female	49.0% (127)	48.9% (85)	49.4% (42)	>0.999
**Risk factors**				
- Hypertension	68% (176)	64.9% (113)	74.1% (63)	0.157
- Atrial fibrillation	40.2% (104)	37.9% (66)	44.7% (38)	0.345
- Diabetes	18.5% (48)	19.0% (33)	17.6% (15)	0.866
- Hyperlipidemia	54.3% (140)	53.4% (93)	56.0% (47)	0.790
Admission NIHSS	11 (6–16)	11 (6–16)	11 (6–17)	0.947
Multiple occlusions on admission	5% (13)	4.6% (8)	5.9% (5)	0.763
Embolic infarcts outside hypoperfused areas	13.1% (34)	12.6% (22)	14.1% (12)	0.845
Site of occlusion				0.680
Posterior circulation	15.1% (39)	13.2% (23)	18.8% (16)	
iICA / Carotid-T	17.8% (46)	19.5% (34)	14.1% (12)	
M1 proximal	22.8% (59)	22.4% (39)	23.5% (20)	
M1 distal	22.8% (59)	23.6% (41)	21.2% (18)	
M2	19.3% (50)	19.5% (34)	18.8% (16)	
M3/M4/A1/A2	2.3% (6)	1.7% (3)	3.5% (3)	
IV tPA	45.2% (117)	43.7% (76)	48.2% (41)	0.509
DWI ASPECTS	8 (7–9)	8 (7–9)	8 (7–10)	0.921
TOAST				0.142
Large artery atherosclerosis	12% (31)	14.4% (25)	7.1% (6)	
Cardioembolism	40.5% (105)	36.2% (63)	49.4% (42)	
Other determined cause	8.1% (21)	8.6% (15)	7.1% (6)	
Unknown cause/multiple causes	39.4% (102)	40.8% (71)	36.5% (31)	
Cardioembolism vs other	40.5% (105)	36.2% (63)	49.4% (42)	0.045*
Treatment group				0.188
Stent-retriever thrombectomy	84.6% (219)	87.4% (152)	78.8% (67)	
Intra-arterial thrombolysis	10.8% (28)	8.0% (14)	16.5% (14)	
Manual fragmentation	0.4% (1)	0.6% (1)	0% (0)	
No intervention	4.2% (11)	4.0% (7)	4.7% (4)	
Cervical/intracranial access				0.127
Distal aspiration	36.3% (94)	37.4% (65)	34.1% (29)	
Balloon-guide catheter	40.5% (105)	42.5% (74)	36.5% (31)	
Distal aspiration and balloon guiding catheter as initial setup	1.9% (5)	2.9% (5)	0% (0)	
Distal aspiration and balloon guiding catheter as secondary setup	1.9% (5)	1.7% (3)	2.4% (2)	
Only non-balloon-guide catheter	19.3% (50)	15.5% (27)	27.1% (23)	
Aortic arch anatomy				
Supraaortal branch stenosis	33.1% (85/257)	30.8% (53/172)	34.1% (29/85)	0.670
Aortic arch elongation				0.141
Type I	37.4% (96/257)	33.1% (57/172)	45.9% (39/85)	
Type II	22.2% (57/257)	23.3% (40/172)	20.0% (17/85)	
Type III	40.5% (104/257)	43.6% (75/172)	34.1% (29/85)	
Aortic arch configuration				0.578
Normal	72.0% (185/257)	73.8% (127/172)	68.2% (58/85)	
Bovine	23.7% (61/257)	21.5% (37/172)	28.2% (24/85)	
SOCCA	3.5% (9/257)	3.5% (6/172)	3.5% (3/85)	
Aberrant subclavian artery	0.8% (2/257)	1.2% (2/172)	0% (0/85)	
Pre-interventional diagnostic angiography only on affected hemisphere	13.1% (34)	14.9% (26)	9.4% (8)	0.245
Number of maneuvers	1 (1–2)	1 (1–2)	1 (1–2)	0.328
Procedure duration (min)	50 (28–86)	47 (28–81)	63 (30–97)	0.158
Cervical stenting	9.7% (25)	11.5% (20)	6.0% (5)	0.184

INT, infarcts in new territory; IINHT, infarcts in initially non-hypoperfused territory; iICA, intracranial ICA; ASPECTS, Alberta Stroke Program Early Computed Tomography Score; TOAST, Trial of Org 10172 in Acute Stroke Treatment; Bovine, common aortic origin of the left CCA and brachiocephalic trunc; SOCCA, separate origin of the left CCA from the brachiocephalic trunk.

### Sensitivity analysis regarding IV tPA administration and its timing

The presence of INT/IINHT was not associated with IV tPA pretreatment status, even when different endpoints and subgroups where considered (i.e. INT only, IINHT only, at least one type A INT/IINHT, at least one size type III INT/IINHT, see Table 2). Notably, comparable Odds Ratios were found when considering only patients treated with stent-retriever thrombectomy (Table 2). The likelihood of developing INT/IINHT were also similar among patients not receiving IV tPA, patients receiving IV tPA with bolus start to procedure initiation ≥70 min (i.e. no therapeutic concentration can be anticipated), and patients undergoing endovascular treatment <70 minutes after IV tPA bolus administration (i.e. therapeutic concentrations can be anticipated, see Supplementary Table e5).

**Table 2:** Crude and adjusted Odds Ratios of IV tPA for different INT/IINHT endpoints.

Cohort	Outcome	Crude Odds Ratio (IV tPA)	Adjusted Odds Ratio
Complete Cohort	INT/IINHT	1.20 (0.71–2.0)	1.383 (0.793–2.412)
Complete Cohort	INT	1.13 (0.66–1.94)	1.324 (0.74–2.37)
Complete Cohort	IINHT	1.89 (0.98–1.11)	2.23 (0.71–7.04)
Complete Cohort	Type A INT/IINHT	0.976 (0.517–1.843)	1.09 (0.55–2.15)
Complete Cohort	Size Type III INT/IINHT	1.744 (0.54–5.65)	2.135 (0.60.-7.51)
Stent-Retriever only	INT/IINHT	1.31 (0.74–2.33)	1.42 (0.78–2.58)
Stent-Retriever only	INT	1.24 (0.68–2.25)	1.40 (0.74–2.63)
Stent-Retriever only	IINHT	1.94 (0.62–6.14)	1.98 (0.57–6.82)
Stent-Retriever only	Type A INT/IINHT	0.92 (0.45–1.89)	0.99 (0.47–2.09)
Stent-Retriever only	Size Type III INT/IINHT	1.43 (0.42–4.82)	1.59 (0.43–5.81)

Adjusted for age, sex, stroke etiology, treatment group and type of protection (right column).

INT, infarcts in new territory; IINHT, infarcts in initially non-hypoperfused territory; IV tPA, thrombolysis with intravenous tissue-type plasminogen activator.

### Clinical relevance

Follow-up results were available for 241/259 patients (93.1%). Patients with INT/IINHT had lower rates of functional independence at day 90 (mRS 0–2, 57.7% vs 71.8%, p = 0.040, Supplementary Figure e4) and higher prevalence of neurological worsening (16.7%, vs 7.7%, P = 0.049). This association persisted when analyses were confined to patients with IINHT without INT vs. controls (mRS 0–2 25.0% vs 71.6%, P = 0.01 and neurological worsening 55.6% vs 7.7%, P < 0.001).

**Figure 5:**
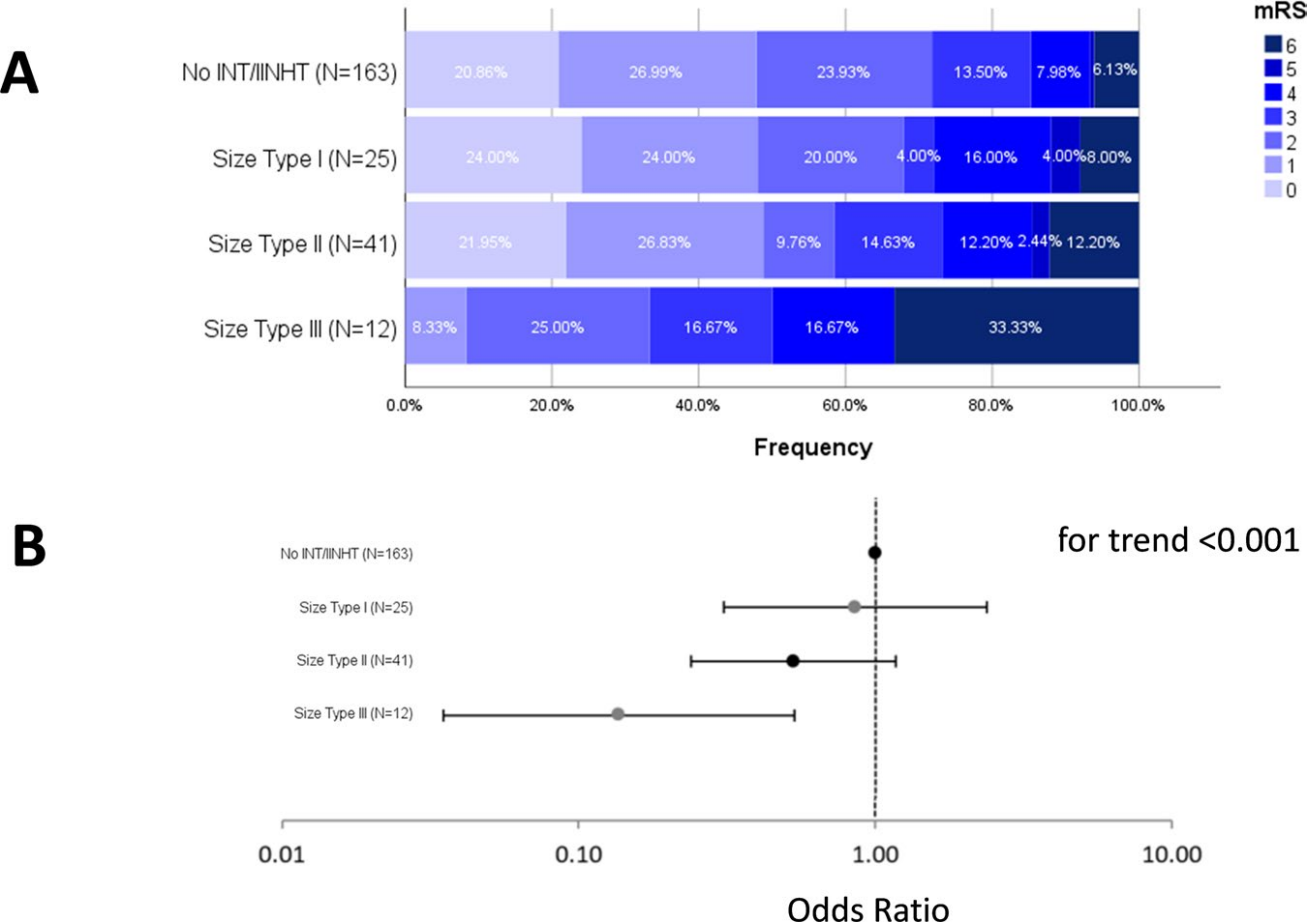
Outcome of patients with strata of INT/IINHT and their size. (**A)** Increased INT/IINHT size was associated with worse outcome; for classification purposes patients with multiple INT/IINHT received the class according to their largest size. (**B)** Odds Ratios for mRS 0–2 (comparator: no INT/IINHT) derived from binary logistic regression with functional independence as dependent variable (cf. methods for covariates in the model). P for trend was evaluated using an extended Mantel Haenszel chi-square for linear trend (one degree of freedom) including a continuity correction. The adjusted Odds Ratio per size increment was aOR 0.63, 95% CI 0.46–0.86.

Compared to patients without INT/IINHT, INT/IINHT patients showed a consistent trend toward poorer outcome with respect to increasing size type (Fig. 5). Implemented as a step-wise categorical variable ranging from the control group (no INT/IINHT, category 0) to at least one size type III infarct (category 3), increasing INT size was associated with lower functional independence (mRS 0–2) after adjusting for age, sex, NIHSS on admission, IV tPA, reperfusion success, DWI-ASPECTS and site of occlusion (aOR 0.63, 95% CI 0.46–0.86). A step-wise increase in INT/IINHT size was associated with neurological worsening, using the same covariates as in the model above (aOR 1.89, 95% CI 1.26–2.86).

## Discussion

The main findings of this observational study were: (1) INT/IINHTs are not rare. They occur in about one-third of patients undergoing endovascular stroke treatment and some patients have multiple INT in different territories. (2) Most INT measure <2 cm and mostly occur in territories where no guiding or microcatheter manipulation was performed across the INT territory orifices (manipulation type B). (3) INT/IINHT are clinical relevant depending on their size. (4) Risk factors seem to include treatment without flow manipulative protection devices and a cardioembolic origin of the stroke. (5) Endovascular treatment without IV tPA does not seem to increase the risk of developing INT/IINHTs.

### Prevalence

We found a higher frequency of INT/IINHTs than reported in previous studies^3,13^. MRI with DWI has shown a much higher sensitivity than CT for detecting acute ischemia and our results show that most INT/IINHTs are smaller than 2 cm, underlining the need to distinguish between CT- and DWI-defined INT rates. Ganesh *et al*. reported 14 INT in 308 patients, resulting in a prevalence of 4.5%. However, only 59 patients were evaluated using MRI with DWI, and here the detection rate was more than doubled (11.7%)^3^. Although this is still considerably lower than the INT/IINHT prevalence that we observed, there is considerable uncertainty due to the small sample size. Additionally, the following factors may have contributed to this discrepancy. First, more than 80% of the patients included in our cohort received pre-interventional diagnostic angiography in territories unrelated to the affected territory, thus giving rise to emboli associated with diagnostic angiography. Second, patient selection criteria in the ESCAPE trial were strict whereas there is a high chance of including older people with more vascular comorbidities in our cohort, potentially complicating angiography and resulting in more emboli.

Importantly, most INT/IINHTs were angiographically occult, corroborating findings on distal embolization during endovascular stroke treatment^14^ and lending further support to INT as a potentially more sensitive technical safety endpoint than angiographically observed ENT.

### Justification for the introduction of IINHT

In the analyses presented, we have introduced another type of new infarct, namely infarcts in initially not hypoperfused territory. Goyal *et al*. defined INT as infarcts in a previously unaffected territory, based on individual anatomy^2^. We agree that, for example, anterior cerebral artery emboli after treatment of an intracranial carotid-terminus occlusion are not emboli in a new territory, because they are located in close anatomic proximity and directly distal to the occlusion site. However, such emboli may be the reason for infarcts occurring in a territory where they would most likely not have occurred if no treatment had been performed (i.e. when this territory was not hypoperfused initially) and are thus different from emboli in the territory of initial hypoperfusion. Another problem is that the final TICI score will only take into account emboli or residual perfusion deficits in the territory of initial hypoperfusion (the so-called ‘target territory’). Therefore, infarcts or emboli in initially not hypoperfused territories that occur directly distal to the occlusion site will not be considered as INT (according to their strict definition), nor will they affect (i.e. reduce) the final TICI score. As a result, we introduced the term “infarct in an initially not hypoperfused territory” (IINHT), which we consider potentially harmful emboli, and a subcategory of new infarcts. Adding to their theoretical relevance, IINHTs were associated with neurological worsening and lower levels of functional independence, even when patients with INT were excluded.

### Timing

We defined INT/IINHT as new infarcts following careful comparison of pre- and post- interventional imaging. As post-interventional imaging was not routinely performed immediately after the procedure, it is uncertain exactly when these new infarcts occurred. Besides the possibility of being related to the interventional procedure, new embolic events may arise from the primary source of the initial large vessel occlusion (e.g. unstable cardiogenic thrombus or ruptured carotid-plaque). Although we were unable to determine the direct cause for INT/IINHT within the current study design, there are several arguments for and against the two possible main mechanisms (Table 3).

**Table 3:** Considerations regarding the potential cause of INT/IINHT.

Arguments in favor of being related to the procedure	Arguments against being related to the procedure
If the index event is caused by an unstable thrombus or plaque, which causes subsequent infarction to other territories, patients with several emboli or multiple occlusions on admission should be more likely to develop INT/IINHT.	Ganesh *et al*. found a comparable frequency of INT in patients treated with IV tPA alone and those additionally treated with mechanical thrombectomy^3^.
If INT are unrelated to the procedure, they can be expected to occur randomly in intracranial territories. However, the percental occurrence of type A manipulation types was close to 50%, thus clearly over-representing territories with a close proximity to the occlusion site and where catheter manipulation took place.	INT also occurred in patients receiving only diagnostic angiography and thus not all infarcts can be associated with the interventional part of angiography (although occurrence due to diagnostic angiography is possible).
Theoretically, a prolonged time interval between the endovascular procedure and follow-up MRI should extend the period during which INT unrelated to the procedure could occur. However, no such association was noted.	

### Associated factors and the role of IV tPA

We observed a higher frequency of INT/IINHT in patients in whom no balloon-guide catheter or distal access catheter was used during the procedure, in line with previous reports^15^, and a higher frequency of INT in patients with a cardioembolic stroke origin. The association between INT and cardioembolic origin seems reasonable, as most INT were classified as non-procedure-related and thus potentially derived from a systemic source, while IINHT are by definition related to the procedure itself.

No association was observed between simple aortic arch elongation metrics, branching patterns, and likelihood of INT/IINHT occurrence. Furthermore, we were unable to reproduce the findings by Ganesh *et al*. and Goyal *et al*., who reported a reduced risk of INT in patients receiving IV tPA before endovascular treatment^3,6^. We performed several sensitivity analyses regarding the timing of IV tPA and different INT/IINHT definitions, but we were unable to detect an association. We cannot be sure, that our cohort was adequately powered to detect a potential treatment effect of IV tPA and confinement to patients with early follow-up MRI may have led to selection bias. In the future, randomized controlled trials including SWIFT-DIRECT, MR CLEAN-NO IV, SKIP, and the DIRECT MT trial may conduct secondary analyses to provide definitive evidence as to whether pre-interventional IV tPA can offer additional benefits regarding the occurrence of INT.

### Strength and limitations

This study provides insight into the prevalence of new DWI lesions in patients undergoing endovascular treatment for large vessel occlusion strokes. A particular strength is the high diagnostic sensitivity of DWI for detecting ischemic lesions while ensuring high specificity by using a comparison to the pre-interventional lesion load. A possible limitation is that confinement to post-procedural MRI may carry the risk of selection bias, as patients who had worse outcomes (unstable, severe moving artifacts) may have been more likely to have a follow-up CT rather than MRI. Hence, there is a risk that the incidence of severe, clinically apparent INT (e.g. large ACA infarcts) was underestimated. In addition, some patients were examined using e.g. 1.5T post-interventionally and 3T pre-interventionally or vice versa, potentially leading to some uncertainty regarding the sensitivity of DWI according to magnetic field strength^16^. Furthermore, the generalizability of our results is limited as not all institutions perform diagnostic angiography before an intervention, which may affect the occurrence of INT. Moreover, some of the patients included were treated with older treatment modalities only. Finally, aspiration-only techniques were not used, and their application might have affected the occurrence of INT/IINHTs.

## Conclusion

INT and IINHT are not rare, often angiographically occult, and may serve as a surrogate endpoint for safety evaluation of new devices and endovascular techniques in the future. Neurological worsening and functional deficits associated with INT and IINHT increase in severity according to their size, and further research on factors associated with such infarcts is warranted.

## Supplementary information

Supplementary information

## Data Availability

Raw data on this cohort can be made available upon request to the corresponding author and after clearance by the local ethics committee.
