# Supplementary material for: Infarct in new territory after endovascular stroke treatment: A diffusion-weighted imaging study
Source: Sci Rep. 2020 May 20;10:8366. doi: 10.1038/s41598-020-64495-2 (PMC7239894; doi:10.1038/s41598-020-64495-2)
Supplement: Supplementary file 1 — Supplementary information [file 41598_2020_64495_MOESM1_ESM.docx]

**Infarct in new territory after endovascular stroke treatment: A diffusion-weighted imaging study**

Johannes Kaesmacher MD^1,2,3*^, Christoph Kurmann MD^1*^, Noel Jungi Medical Student^2^, Philipe Breiding MD^1^, Matthias F. Lang MD^1,3^, Raphael Meier PhD^4^, Tomas Dobrocky MD^1^, Eike Piechowiak MD^1^, Felix Zibold MD^1^, Sebastian Bellwald MD^1^, Thomas R. Meinel^1^, Mirjam R Heldner^1^, Pasquale Mordasini MD^1^, Marcel Arnold MD^1^, Pascal J. Mosimann MD^1^, Mayank Goyal MD^5^, Jan Gralla MD^1^* & Urs Fischer MD^2^*

* These authors contributed equally to the manuscript

**1** University Institute of Diagnostic and Interventional Neuroradiology, University Hospital Bern, Inselspital, University of Bern, Bern, Switzerland

**2** Department of Neurology, University Hospital Bern, Inselspital, University of Bern, Bern, Switzerland

**3**Department of Diagnostic, Interventional and Pediatric Radiology, University

Hospital Bern, Inselspital, University of Bern, Bern, Switzerland

**4** Support Center for Advanced Neuroimaging - Institute for Diagnostic and Inter-ventional Neuroradiology, University Hospital Inselspital and University of Bern, Bern, Switzerland

**5**Department of Diagnostic Imaging, University of Calgary, Calgary, Alberta, Canada

*Contributed equally

**Supplemental Data**

Supplementary Tables: 5

Supplementary Figures: 4

**Supplementary Tables**

**Table e-1** – Standardized Readers Worksheet

| Good quality DWI for diagnostic use: |  |
| --- | --- |
| - Preinterventional | **□yes □no** |
| - Postinterventional | **□yes □no** |
| Pre-interventional MRP with good quality for diagnostic use: | **□yes □no** |
| Multiple distinct perfusion deficits (e.g. P1 left, M2 right) suggestive for multiple occlusions: | **□yes □no** |
| DWI lesions outside hypoperfused areas on pre-interventional images | **□yes □no** |
| Infarcts in new territories (INT) according to definition by Goyal et al. | **□yes □no** |
| Total number of INTs |  |
| *INT 1* |  |
| - Location | **□left □right □midline** |
| - Territory | **□ PICA □ AICA □SUCA □VA/BA □PCA □MCA □ACA** |
| - Size according to Goyal et al. | **□1 □2 □3** |
| - Type of Manipulation according to Goyal et al. | **□A □B** |
| *INT 2* |  |
| - Location | **□left □right □midline** |
| - Territory | **□ PICA □ AICA □SUCA □VA/BA □PCA □MCA □ACA** |
| - Size according to Goyal et al. | **□1 □2 □3** |
| - Type of Manipulation according to Goyal et al. | **□A □B** |
| *INT 3* |  |
| - Location | **□left □right □midline** |
| - Territory | **□ PICA □ AICA □SUCA □VA/BA □PCA □MCA □ACA** |
| - Size according to Goyal et al. | **□1 □2 □3** |
| - Type of Manipulation according to Goyal et al. | **□A □B** |
| *INT 4* |  |
| - Location | **□left □right □midline** |
| - Territory | **□ PICA □ AICA □SUCA □VA/BA □PCA □MCA □ACA** |
| - Size according to Goyal et al. | **□1 □2 □3** |
| - Type of Manipulation according to Goyal et al. | **□A □B** |
| *INT 5* |  |
| - Location | **□left □right □midline** |
| - Territory | **□ PICA □ AICA □SUCA □VA/BA □PCA □MCA □ACA** |
| - Size according to Goyal et al. | **□1 □2 □3** |
| - Type of Manipulation according to Goyal et al. | **□A □B** |
| Infarcts in initially non-hypoperfused territory (IINHT), e.g. ACA in Carotid-T occlusions with collateralization via AcomA) |  |
| Total number of IINHTs |  |
| *IINHT 1* |  |
| - Location | **□left □right □midline** |
| - Territory | **□ PICA □ AICA □SUCA □VA/BA □PCA □MCA □ACA** |
| - Size according to Goyal et al. | **□1 □2 □3** |
| - Type of Manipulation according to Goyal et al. | **□A □B** |
| *IINHT 2* |  |
| - Location | **□left □right □midline** |
| - Territory | **□ PICA □ AICA □SUCA □VA/BA □PCA □MCA □ACA** |
| - Size according to Goyal et al. | **□1 □2 □3** |
| - Type of Manipulation according to Goyal et al. | **□A □B** |
| *IINHT 3* |  |
| - Location | **□left □right □midline** |
| - Territory | **□ PICA □ AICA □SUCA □VA/BA □PCA □MCA □ACA** |
| - Size according to Goyal et al. | **□1 □2 □3** |
| - Type of Manipulation according to Goyal et al. | **□A □B** |
| *IINHT 4* |  |
| - Location | **□left □right □midline** |
| - Territory | **□ PICA □ AICA □SUCA □VA/BA □PCA □MCA □ACA** |
| - Size according to Goyal et al. | **□1 □2 □3** |
| - Type of Manipulation according to Goyal et al. | **□A □B** |

**Table e-2** – Infarct to new territory classification according to Goyal et al.^1^

| Classification based on size | | Classification based on catheter manipulation across territory ostium | |
| --- | --- | --- | --- |
| **Type I** | ≤2mm diffusion lesion (unidentifiable on non-contrast CT scan) | **Type A** | Catheter was manipulated past the ostium of the new territory (e.g. large ACA infarct in a patient with an initial M1 occlusion): greater likelihood that infarct is related to the procedure |
| **Type II** | >2mm to ≤20mm lesion (could potentially be difficult to identify on CT scan) | **Type B** | Catheter was not manipulated past the ostium of the new territory (e.g. left PICA infarct in a patient with an initial right M1 occlusion): lower likelihood that infarct is related to procedure |
| **Type III** | Large (>20 mm) infarct in new territory |  |  |

**Table e-3** – Relation between INT/IINHTs and angiographically visible emboli

| **A** |  | INT/IINHT | |  |
| --- | --- | --- | --- | --- |
|  |  | + | - | Total |
| Emboli in post-procedural angiography overview runs | + | 8 | 3 | 11 |
|  | - | 74 | 167 | 242 |
|  | Total | 82 | 171 | 253* |
| INT, Infarct to new territory; IINHT, Infarcts to initially non-hypoperfused territory; *, total N of patients was reduced from 259 to 253 because good-quality post-procedural angiography runs were only available for 253 patients (cf. Methods). | | | | |

| **B** |  |  | | |  |  |
| --- | --- | --- | --- | --- | --- | --- |
|  |  | At least one type size III | At least one type size II (but not III) | At least one type size I (but no II/III) | No INT/IINHT | Total |
| Emboli in post-procedural angiography overview runs | + | 2 | 5 | 1 | 3 | 11 |
|  | - | 10 | 40 | 24 | 168 | 242 |
|  | Total | 12 | 45 | 25 | 171 | 253* |
| INT, Infarct to new territory; IINHT, Infarcts to initially non-hypoperfused territory; *, total N of patients was reduced from 259 to 253 because good-quality post-procedural angiography runs were only available for 253 patients (cf. Methods). | | | | | | |

**Table e-4** – Stroke origin in patients with INT, IINHT and controls

|  | All | INT | IINHT (excluding those with concomitant INT) | Patient without INT/IINHT | P |
| --- | --- | --- | --- | --- | --- |
| TOAST |  |  |  |  | 0.009 |
| - Large artery atherosclerosis | 12% (31/259) | 3.9% (3/76) | 33.3% (3/9) | 14.4% (25/174) |  |
| - Cardioembolism | 40.5% (105/259) | 53.9% (41/76) | 11.1% (1/9) | 36.2% (63/174) |  |
| - Other determined cause | 8.1% (21/259) | 6.6% (5/76) | 11.1% (1/9) | 8.6% (15/174) |  |
| - Unknown cause / multiple causes | 39.4% (102/259) | 35.5% (27/76) | 44.4% (4/9) | 40.8% (71/174) |  |

**Table e-5** – Relation between INT/IINHTs and angiographically visible emboli

|  | Patients not receiving IV tPA | Patients receiving IV tPA with bolus start ≥70min procedure begin | Patients receiving IV tPA with bolus start <70min procedure begin | Total |  |
| --- | --- | --- | --- | --- | --- |
| INT/IINHT | 31.0% (44/142) | 33.3% (16/48) | 36.2% (25/69) | 85 |  |
| No INT/IINHT | 69.0% (98/142) | 66.7% (32/48) | 63.8% (44/69) | 174 |  |
| Total | 142 | 69 | 48 | 259 |  |
|  |  |  |  |  | P=0.746 |

**Supplementary Figures**

**Figure e-1** **–** Standardized Data Presentation


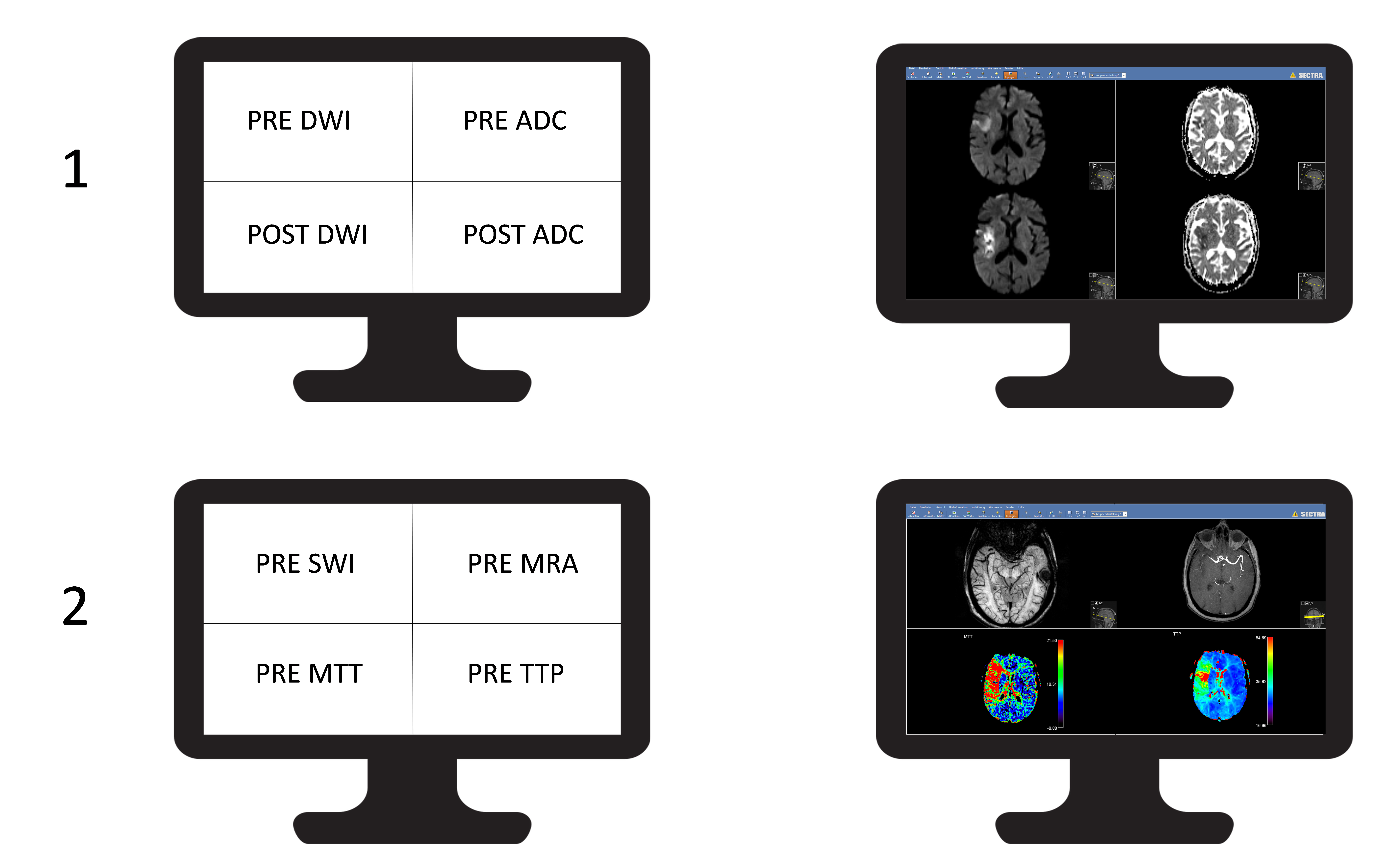


Cases were presented to the readers on the Picture Archiving Communication System (IDS7, Sectra, Linköping, Sweden) Workplaces using a two-monitor set up. PRE, pre-interventional; POST, post-interventional; DWI, diffusion weighted imaging; ADC, apparent diffusion coefficient maps; SWI, susceptibility weighted imaging; MRA, magnetic resonance angiography; MTT, mean transit time maps; TTP, time to peak maps. Perfusion maps were processed using the software package Olea Sphere^®^ (Version 2.3, oscillation index singular value decomposition method and automatically determined arterial input function).

**Figure e-2** **–** Patients with infarct to initially non-hypoperfused territory (IINHT) and infarcts to new territory (INT)

**
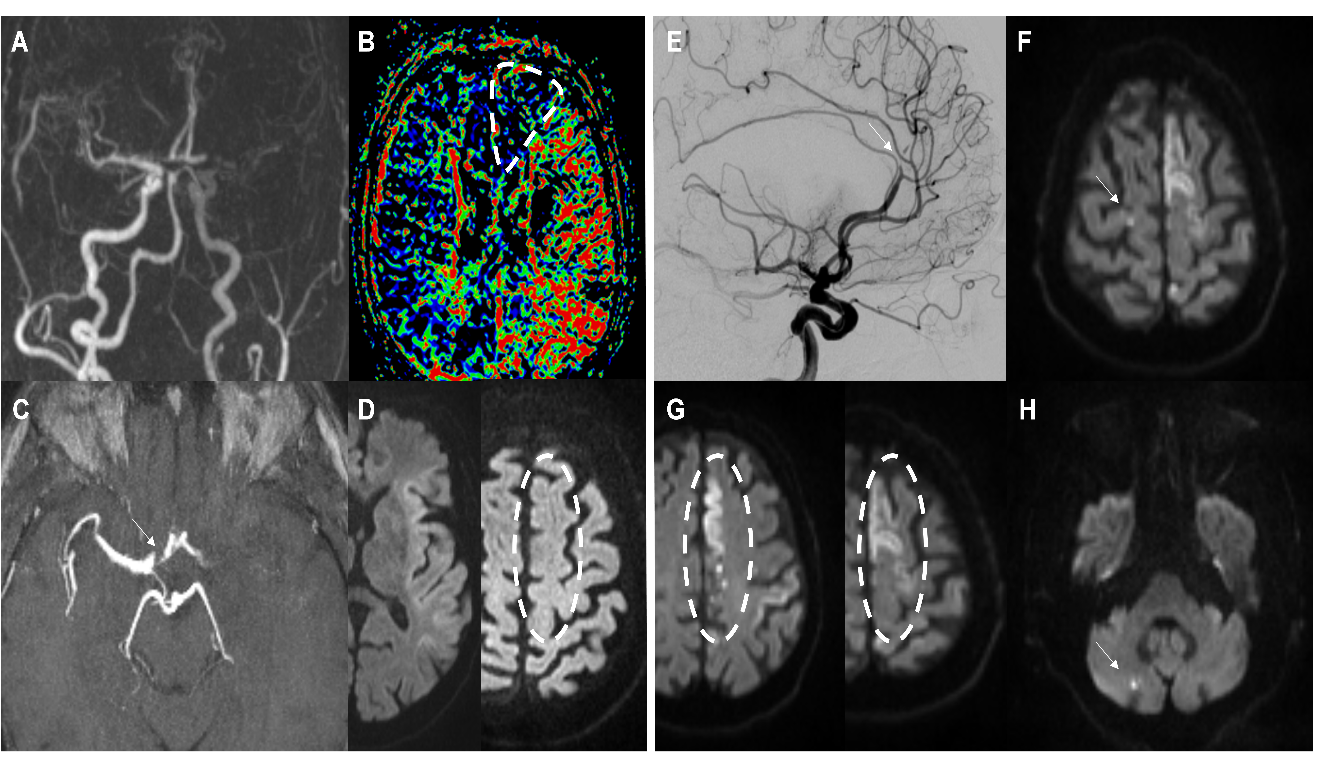
**

**A**, contrast enhanced MR angiography revealed a left-sided Carotid-T occlusion; **B**, mean transit time perfusion maps suggest no perfusion delay in the ipsilateral anterior cerebral artery, likely because antegrade flow is ensured via a small right-sided A1 segment and patent anterior communicating artery (**C**). **D**, faint diffusion restriction within the left-sided MCA territory, without abnormalities within the anterior cerebral artery territory. **E**, peri-interventional anterior cerebral artery embolism, as evidenced by contrast stagnation; **F-H**, Post-interventional DWI reveals punctual infarcts to new territory (INT) in the right sided central region (F, arrow, size type I, manipulation type B INT) and right cerebellar hemisphere (**H**, arrow, size type I, manipulation type B INT), while the DWI slices in **G** display a new large anterior cerebral artery territory infarct, corresponding to a size type III, manipulation type A infarct to initially non-hypoperfused territory (IINHT).

**Figure e-3 –** Patients with multiple occlusions and infarcts outside hypoperfused areas on admission imaging


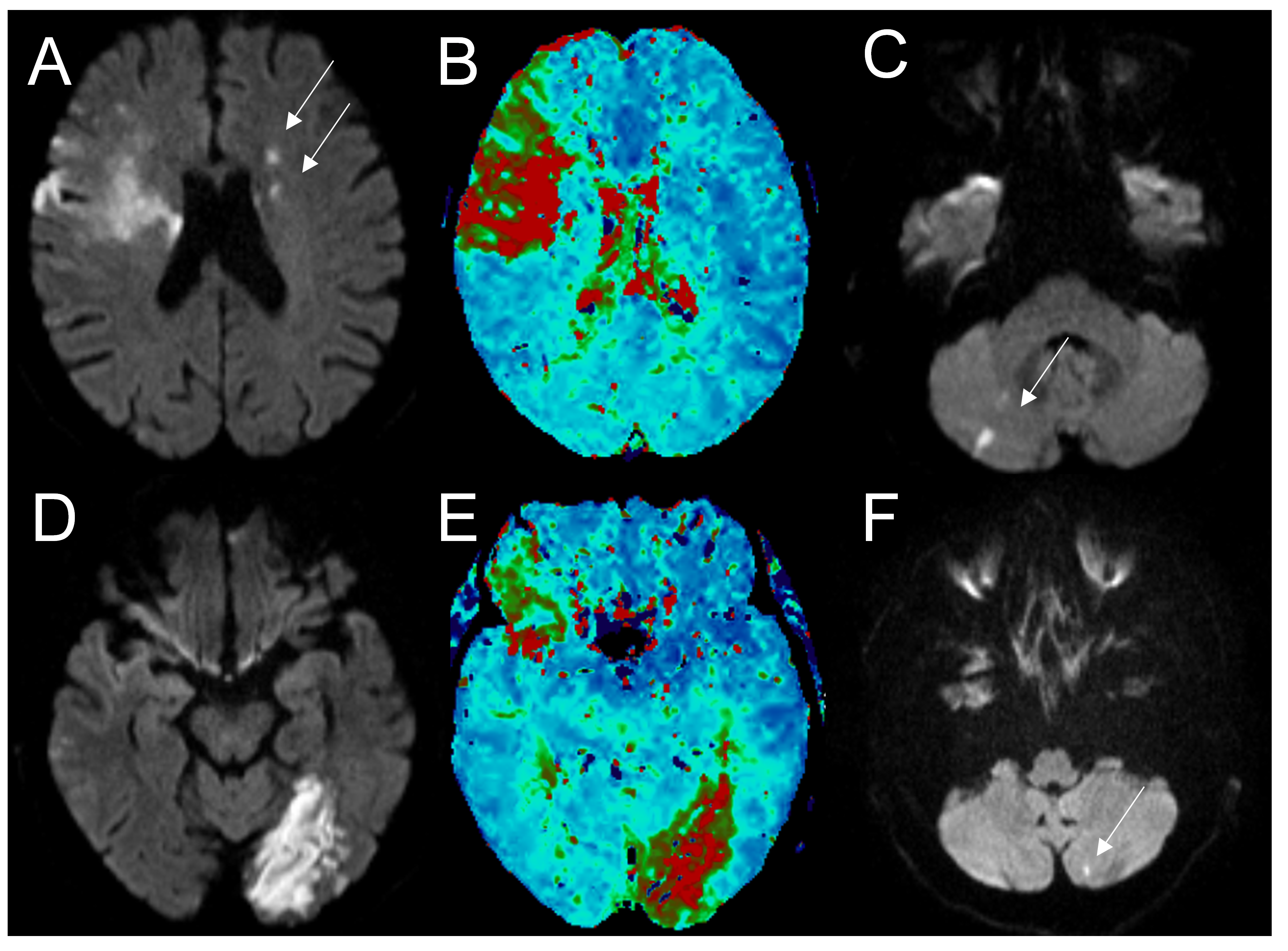


Patient presenting with a right-sided M2 superior trunk division occlusion, together with a left-sided P2 occlusion (A/B, D/E). Besides infarcts confined to the hypoperfused territory, small DWI lesions are seen in the deep white matter of the left sided MCA territory (A, white arrows) and both cerebellar hemispheres (white arrows C/F). After endovascular treatment, no new DWI lesions outside the initially affected territories were found (no INTs).

**Figure e-4 –** 90 days functional outcome in patients with and without INT/IINHT


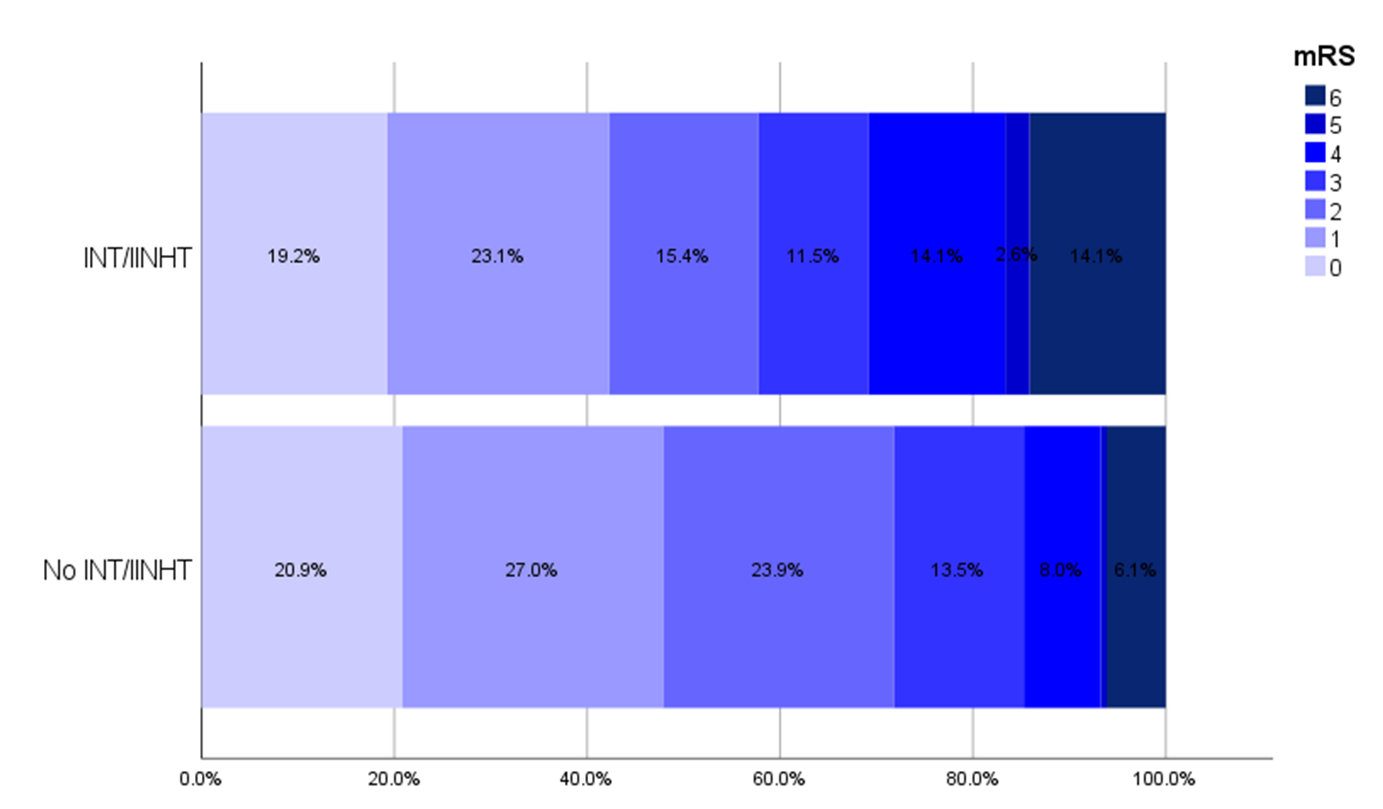
Patients with INT/IINHT had lower rates of functional independence at day 90 (57.7% vs 71.8%, p=0.040).
